# Implementation of robotic surgery in Dubai: a focus on outcomes

**DOI:** 10.1007/s11701-022-01407-8

**Published:** 2022-04-19

**Authors:** Mouhammad Halabi, Jado Kamal, Farida Reguig, Michael E. Zenilman, Hatem Moussa

**Affiliations:** 1 The Department of Surgery, American Hospital in Dubai, Oud Metha, Dubai, UAE; 2 Royal College of Surgeons Ireland – Bahrain, Muharraq, Bahrain; 3 Department of Surgery, Weill Cornell Medicine, New York, NY USA

**Keywords:** Robotic surgery, Robotic surgery program, Inguinal hernia, Cholecystectomy

## Abstract

The rapid acceptance of robotic surgery in gallbladder, inguinal, and ventral hernia surgery has led to the growth of robotic surgery programs around the world. As this is new technology, implementation of such programs needs to be done safely, with a focus on patient outcomes. We herein describe the implementation of a new robotic surgery program in a major hospital in the Middle East. A laparoendoscopic surgeon led the program after training and proctoring. Competency based credentialing were created and put in place. To confirm safety of the program, all laparoscopic and robotic cholecystectomy and hernia operations were followed, and perioperative data analyzed. Out of the 304 patients included in this study, 157 were performed using the robotic approach. In the cholecystectomy group (*n* = 103) the single site approach offered shorter operative times (*P* < 0.05). Both the single site robotic and the robotic assisted approaches resulted in less pain (*P* < 0.05). In the inguinal hernia group (*n* = 146) the laparoscopic approach offered shorter operative times (*P* < 0.05), but the robotic approach was associated with less pain (*P* < 0.05). In the ventral hernia group (*n* = 55), the open approach offered the best operative times, but the robotic approach was associated with the least amount of pain (*P* < 0.05). This is the first report of the implementation of a robotic program in the MENA region where the primary measure of success is outcomes. We show that monitoring cholecystectomy, inguinal or ventral hernia data can confirm the quality of the program before expansion and moving forward to more complex procedures.

**Supplementary Information:**

The online version contains supplementary material available at 10.1007/s11701-022-01407-8.

## Introduction

The expanding use of robotic surgery around the world has resulted in it becoming a more popular surgical option, moving towards the technique of choice for many common procedures [1]. The rapid adoption of new technology should be done with caution to minimize adverse events. This happened during the swift implementation of laparoscopic surgery thirty years ago [2]. While the basis of robotic surgery has been centered in the United States, its worldwide appeal has resulted in the creation of isolated robotic centers in Europe, Asia, and the Middle East.

Several publications have addressed the process of implementing a robotic program [3–6], but few have focused on quality or outcomes. This should be the benchmark of any new program, as patient safety, competency of the surgeons, and efficiency of the team should be measurable standards. Aside from developing novel indications for robotic use, the implementation of a new program should focus on the basics—performance of safe, reproducible results without harm for standard, accepted surgical procedures.

We describe herein the implementation of such a program, employing such standards for the first such program at the American Hospital of Dubai (AHD), United Arab Emirates. We report results for standard cholecystectomy and hernia and used this opportunity to compare the robotic approaches for open, laparoscopic and single site approaches.

## Methods

This study retrospectively reviewed maintained data for patients who underwent hernia and gallbladder surgery between February 2020 and March 2021, performed by a single surgeon (HM) at the American Hospital of Dubai. The surgeon, trained in the United States, is board certified by the American Board of Surgery (Philadelphia, PA). The The DaVinci Robot (Xi—OS4; v9, Intuitive Surgical, Inc., Sunnyvale, California). The study was approved by the AHD Institutional Review Board.

In collaboration with hospital leadership, a strict credentialing process was created to assure safety, based on the privileging criteria established at New York Presbyterian Brooklyn Methodist Hospital, an affiliate of Weill-Cornell Medicine (Supplemental Appendix). Briefly, any surgeon wishing to perform robotic surgery needed to have core privileges, possess active laparo-endoscopic privileges and proof of competency in gallbladder and hernia procedures, complete the online modules and receive a certificate for the specific DaVinci surgical system, complete the simulation training on the Mimic "backpack" simulator and the mandatory simulation modules, attend a hands-on training practicum in use of DaVinci platform and was proctored.

Cholecystectomy was performed via the standard multiport laparoscopic approach, multiport robotically, or by single-site using a bladeless, blunt-tip 8 mm trocar (Intuitive Surgical, Inc., Sunnyvale, California). Inguinal or ventral hernias were repaired open, laparoscopically, or robotically using mesh technique (Progrip self-fixating mesh, Medtronic, Minneapolis, Minnesota). The approach was individualized for each person by the surgeon. As this was a new platform, there was a progression in the complexity of surgeries to the robot.

The following variables were analyzed: age, sex, BMI, past medical history, previous surgeries, total operative time, conversion to open approach, operative and postoperative complications, length of stay (LOS), postoperative pain scale, cosmesis satisfaction. The total operative time for any robotic procedure was defined as console start to console finish, no docking times were included. Length of stay was defined as one day or less (specifically inclusive of an overnight stay) or more than one day in the hospital. Intraoperative complications were assessed using the Clavien–Dindo classification [7].

Postoperative pain was assessed using a standard numerical pain rating scale [8], with 0 being no pain and 10 being the worst imaginable pain. All postoperative pain scores were assessed one day after the completion of any procedure, either by in person or phone interview. For cosmetic satisfaction, it was self-reported 10-point visual analogue scale, with 0 being complete satisfaction and 10 being no satisfaction, which has previously shown to be reliable [9]. Also to note, all patients were followed up for an average 4 weeks ± 1.

All patients in this study were required to be over the age of 18, had to have undergone either cholecystectomy, inguinal hernia repair, or ventral hernia repair between February 2020 to March 2021 by a single surgeon (HM). Any patients that did not fit these criteria were excluded from this study.

Data are presented as mean ± standard deviation. Analysis was performed using the Student’s *t *test for means and Chi-square analysis for categorical variables. Significance was defined as *p* < 0.05.

### Surgical technique

In the cholecystectomy group, both the robotic and multiport laparoscopic groups used four ports for all the procedures mentioned in this series. In the single site group, one umbilical incision was made, after which a silicon ring was placed through the incision. The ring consisted of two curved ports and an assisting port (which was not used for all procedures).

With regards to ventral and inguinal hernias. All laparoscopic and robotic procedures used a three-port placement encompassing the transabdominal preperitoneal approach.

In the ventral hernia group, patients that underwent the open approach had an incision over the hernia site. Once the facial defect was identified, the hernia sac was dissected two to four cm circumferential margin of intact fascia. Abdominal contents were then mobilized back into the abdominal cavity, a mesh was then placed underneath the fascia.

## Results

Table 1a shows the demographics for the procedures performed and their respective approaches. Of the 304 surgeries performed in this series, there were 128 laparoscopic, and 157 robotic approaches; there were only 19 open surgeries, all of them ventral hernia. There were no differences in age ranges or sex ratio for the 101 cholecystectomies, 146 inguinal herniae performed. In the ventral hernia group, the robot group had significantly (*P* < 0.05) more women and younger age. These inconsistencies are likely due to selection bias during the program's initiation.

**Table 1:** Demographics

	Number	Age ± SD	Female	Male
**a Demographics**				
*Cholecystectomy*				
MultiPort laparoscopic	33	44.39 ± 12.18	19	14
Robotic	30	43.7 ± 10.00	20	10
Single-site robotic	40	40.85 ± 11.96	27	13
*Inguinal Hernia*				
Laparoscopic	78	49.39 ± 12.41	74	4
Robotic	68	46.72 ± 11.82	59	9
*Ventral Hernia*				
Laparoscopic	17	49.59 ± 13.06	7	10
Robotic	19	40.74 ± 12.28 *	14	5*
Open	19	47.05 ± 13.88	13	6

	BMI	Past medical history			Ca	Past surgical history		
		HTN	DM	Obesity		Biliary	Prev Abd	Groin
**b Demographics**								
*Cholecystectomy*								
Laparoscopic	28.30 ± 5.10	9	9	0	0	2	10	0
Robotic	29.73 ± 6.26	6	7	11	1	0	9	1
Single-Site Robotic	29.46 ± 4.11	3	6	2	1	3	7	0
*Inguinal Hernia*								
Laparoscopic	26.90 ± 3.80	21	7	13	1	2	11	2
Robotic	27.01 ± 4.15	9	5	13	0	3	11	8
*Ventral Hernia*								
Laparoscopic	31.96 ± 7.90	2	2	6	0	1	2	2
Robotic	29.01 ± 4.93	6	2	7	0	0	11	0
Open	31.49 ± 6.50	4	4	11	1	0	4	1

BMI = body mass index; HTN = hypertension; DM = Diabetes mellitus, Ca = cancer; Biliary- any biliary procedure/instrumentation (includes cholecystectomy and endoscopic retrograde cholangiopancreatography); Prev Ab = any previous abdominal surgery (including Caesarian Section); Groin = inguinal or femoral hernia

*p < 0.05 compared to laparoscopic

Table 1b shows that the body mass indices did not differ between the groups. Comorbidities are outlined in the table as well. Obesity was consistently the most common comorbidity in the ventral hernia group with the open, laparoscopic, and robotic patients, with rates being 57.9%, 35.3%, and 36.8%, respectively.

Past surgical history is shown in Table 1b. In the cholecystectomy patient group, cesarean-section (c-section) was the most frequent in the single site, laparoscopic, and robotic approaches, at 10.0%, 18.2%, and 23.3%, respectively. A history of previous hernia repair was also consistently the most common in the laparoscopic and robotic inguinal hernia patient groups at 7.7% and 8.8%, respectively. In the laparoscopic and robotic ventral hernia groups, c-section was the most common at 11.8% and 26.3%, respectively. In the latter hernia group, 10.5% of patients that underwent the open approach had a previous hernia repair.

Indications for surgery are shown in Table 2. While there was no difference between laparoscopic and robotic approaches, there appeared to be a bias against the single site approach for acute cholecystitis. There also was a trend towards robotic favoring laparoscopic for bilateral inguinal herniae, likely due to the ease of the approach.

**Table 2:** Indications

	Cholecystectomy			
	Number	Chronic	Acute	Polyp
Laparoscopic	33	21	11	1
Robotic	30	18	12	0
Single Site	40	34	5*	1

		Inguinal Hernia			
		Reducible	Incarcerated	Unilateral	Bilateral
Laparoscopic	78	28	5	16	19
Robotic	68	17	5	6	17

		Ventral Hernia	
		Reducible	Incarcerated
Laparoscopic	17	4	8
Robotic	19	4	0
Open	19	6	5

*p < 0.05 compared to robotic

Table 3 shows the operative findings. While the multiport laparoscopic approach was quicker than the robotic, the single site robotic cholecystectomy showed the fastest OR times, statistically shorter than both multiport laparoscopic and robotic approaches. The former was likely due to the learning curve of robotic surgery, and the latter likely due to a bias towards operating on acute cases laparoscopically. In the hernia surgeries, as suspected the robotic approach was statistically longer for when compared to both open and laparoscopic approaches.

**Table 3:** Operative findings

	Number	Avg time	Conversions	Inflammation/Spillage	Porcelain
*Cholecystectomy*					
Laparoscopic	33	64.12 ± 30.83*	1	4	1
Robotic	30	79.87 ± 24	0	5	
Single Site Robotic	40	58.6 ± 24.7**	0	4	
*Inguinal Hernia*					
Laparoscopic	78	60.91 ± 22.77*	1		
Robotic	68	73.63 ± 27.38	0		
*Ventral Hernia*					
Laparoscopic	17	65.00 ± 30.10	0		
Robotic	19	88.16 ± 25.65***	0		
Open	19	35.47 ± 21.99***	0		

Inflam = inflammation; Spillage- spillage of gallbladder contents; Porcelain = Porcelain gallbladder found at surgery

*Compared to robotic, **Compared to laparoscopic, ***Compared to laparoscopic

We observed no major intraoperative complications in any of the patient groups. In the cholecystectomy patient group, six intraoperative complications (Clavien-Dindo grade 1, see supplemental table in Appendix) were recorded; all were due to bile spillage. Of the total six complications in the cholecystectomy group, four occurred in the single site group; this was likely due to the restricted range of motion associated with the approach. Two complications were recorded in the multi-port laparoscopic group, and none were recorded in the robotic group. With regards to the inguinal hernia and ventral hernia groups no intraoperative complications were recorded.

Throughout this entire series, we observed two operative conversions (Table 3). In the cholecystectomy group we observed one conversion from the multiport laparoscopic approach to the open approach in a 47 year old male that presented with acute cholecystitis, he had no major co-morbidities other than a history of hypertension. During the procedure the surgeon found that the gallbladder wall was very hard and thickened, he was unable to use the laparoscopic grabber to gain control of the gallbladder; there was also some concern about neoplastic pathology behind the gallbladder. Both of the aforementioned reasons warranted conversion to the open technique as the surgeon was unable to continue laparoscopically. The patient fully recovered and no postoperative complications were noted. In the Inguinal hernia group, a 64-year-old male with no significant co-morbidities was scheduled for laparoscopic repair of a left inguinal hernia. During the procedure, the surgeon noticed that a large piece of omentum was incarcerated within the left groin going all the way down to the scrotum. Many attempts were made to dissect the omentum, but there was no way to mobilize it safely. Due to this, the surgeon decided to convert the procedure to the open approach. The patient fully recovered with no postoperative complications.

Table 4 shows that the single site approach had the shortest length of stay after cholecystectomy, with a clear majority leaving on the day of surgery 82.5% vs. 58% for laparoscopic and 16% for robotic approach. Most of the hernia surgery patients went home on the day of surgery or postoperative day 1. There were no differences in postoperative pain scores, or readmission rates.

**Table 4:** Outcomes

	Hosp stay		Cosmesis		Pain score	
	0–1	> 1	0	> 0	**≤**3	≥4
*Cholecystectomy*						
Laparoscopic	19	14	10	23	2	31
Robotic	25	5*	8	22	19	11*
Single site	33	7**	28	12*	26	14*
			**≤3**	**≥4**		
*Inguinal Hernia*						
Laparoscopic	75	3	47	31	11	67***
Robotic	63	5	40	28	48	20
*Ventral Hernia*						
Laparoscopic	15	2	13	4	2	15
Robotic	19	0	15	4****	16	3****
Open	15	4	1	18	0	19

*Compared to laparoscopic

**Compared to laparoscopic and robotic

***Compared to robotic

****Compared to Laparoscopic and Open

There were no major postoperative complications in our entire series. In the cholecystectomy group, one patient (from the single site group) returned to the emergency department one week post-op complaining of right upper quadrant pain, the investigation was negative, and the patient later discharged. One patient from the laparoscopic group also presented to the emergency room 5 days post-operatively with elevated liver function tests, which subsequently normalized. None of the patients in the inguinal hernia group had any post-op complications. While in the open ventral hernia group, one patient experienced a recurrence six months later, those who underwent laparoscopic and robotic procedures did not have any post-op complications. Careful selection resulted in outcomes that were consistent with safe surgery.

Table 4 shows that postoperative pain scores were favorable to the robotic approach in cholecystectomy, inguinal and ventral hernia.

Patient reported surveys confirmed that they were satisfied with the cosmetic results. As expected, the single site, laparoscopic and robotic approaches were favored over open, and single sites were favored over multiport and open surgery (Table 4).

## Discussion

In this series, we describe the implementation of a new, and ultimately safe robotic program in a major hospital in the Middle East. Importantly, our results are consistent with other investigators, who have shown that robotic surgery can be performed effectively for gallbladder, inguinal hernia, and ventral hernia surgery [9–11].

Launching any new surgical platform is inherently risky; institutional dedication toward ensuring physician and operational oversight with a focus on outcomes should be the norm. Too rapid an implementation without confirming proficiency will result in complications, as was observed after the rapid implementation of laparoscopic cholecystectomy in the 1990s [2]. We instituted the current program with an experienced minimally invasive surgeon, implemented a strict credentialling program and proved safe outcomes. We also used our data to compare the robotic approach to both laparoscopic and open procedures, which created an academic atmosphere by interrogating, reviewing and reporting our own data. Lastly, we made an effort to include patient reported outcomes as part of the dataset. This model of investigative implementation should be adopted by any new program.

Our cholecystectomy results are in line with other previous studies [12, 13], with no significant complications, and negligible conversion rates. We did note an unexpected finding in that single site robotic procedures had the shortest OR time, which was not consistent with other reports [13, 14]. This is likely due to a selection bias (described in the Results section) and our reporting technique, in that we did not include the docking and undocking times of the robot. We also found that robotic and single site patients experienced a shorter length of stay and less postoperative pain than laparoscopic, consistent with what was reported by others [12, 15]. Our findings are also consistent with by Kudsi et al. and Pietrabissa et al. [13, 16] who demonstrated that single site approach was significantly associated with better cosmetic outcomes. While we did not observe any postoperative port-site hernias, which have been reported with that approach [17], this study did not follow patients longer than a few months.

Our inguinal hernia results showed that when compared to laparoscopic, the robotic approach had significantly longer operative times, negligible conversion rates, and no significant differences in length of stay. This is in line with other studies [18]. Importantly, our results also agree with the RIVAL trial, which showed no difference between the readmission rates and cosmetic satisfaction of the robotic approach when compared to the laparoscopic [19]. We also noted lower postoperative pain scores in the robotic group, in agreement with the study by Waite et al. [20] though their study reported longer operative times in the robotic approach.

Our ventral hernia group also showed negligible readmission and conversion rates, the robotic approach showed the longest operative time, similar to findings were previously reported by Olavarria et al. [21]. With regards to cosmetic satisfaction, a previous study by Misiakos et al. noted the superiority of the laparoscopic intervention over the open intervention [22], which was confirmed in our findings. Moreover, we found that the robotic approach was superior to open in terms of cosmetic satisfaction as well. When assessing postoperative pain, we found that the RA approach was associated with significantly less pain when compared to the open and laparoscopic groups. This finding was previously reported in a study by Lindström et al. They explained that the reason behind the robotic being associated with less pain might be due to the atraumatic dissection related to the ergonomics of the robot; however, further studies are needed to confirm this finding [23].

We, therefore, confirm that for gallbladder, inguinal, and ventral hernia, our results are consistent with other centers with respect to all measurable parameters.

When the American Hospital in Dubai decided to create the first robotic surgery program in the region, we advised the administration that pivotal for success was an experienced laparoendoscopic surgical leader. Our lead surgeon, by understanding of the local culture and hospital logistics, was able to enlighten the hospital administration, referring physicians and most importantly, the patients on the use and benefits of robotic surgery. Through partnership with the industry, he obtained training, proctoring, and expertise to create a dedicated team. We believe that understanding patient culture, as well as the guidelines put in place for patient selection, was the main driving force in the success of our program. Our data confirm that in the hands of experienced surgeons and leadership, advanced surgery and novel approaches can be introduced with minimal complications.

As no training center was available locally, a barrier to any program, we created strict criteria for credentialing used by an American hospital (Supplemental Appendix). This foundation allowed the American Hospital to create policies and guidelines to be able to train robotic surgeons and pave the way for them to practice independently. We currently have over ten surgeons trained, proctored, and actively performing robotic surgery. There now exists a quality-driven program with case reviews and presentations. As our program expands to novel robotic platforms, these processes will drive the quality.

Our study is retrospective and has important limitations which should be outlined. As noted in the results section, there was selection bias, as evidenced by the choice of surgeries for ventral hernia and single-site surgical approach. An example is that in the ventral hernia group, patients who had previous biliary surgery were more likely to undergo the open approach rather than the laparoscopic or robotic. This is likely due to selection bias, likely to avoid adhesions. Bias was also evident in the inguinal hernia group; the robotic approach was more favored for patients with bilateral inguinal hernia, due to the ease of the approach. We did accept selection bias as part of the safe growth of the program. Also, since this manuscript presents data from a single surgeon, it limits our ability to generalize the results to the entire population. We had to keep our inclusion/exclusion criteria as broad as possible, in order to obtain a reasonable amount of patients for analysis. This has introduced the heterogeneity in our data and may have led to confounding. Last, while we did employ patient reported outcomes, we did not include detailed patient survey data; this will be included in future studies.

The success of our program has allowed our hospital to be the destination center for robotic surgery in the Middle East and North Africa Region. Currently, to our knowledge, we have the highest volume of patients undergoing robotic surgery in the region. We are also the only hospital in the region to offer single site robotic cholecystectomy.

We have demonstrated how to create a new program in a major hospital in the Middle East; we expect it will lead to enhanced surgical care for patients in the Mid-East/North Africa region of the world. Importantly the foundation is set to help create other new programs in the region, and to add novel approaches for surgery. In recent years, many new robotic platforms have come to market. In the MENA region, the Cambridge Medical Robotic (CMR) surgical system has recently gained popularity. With that being said, our hospital has acquired the CMR surgical system; because of this, we believe that our hospital may possibly serve as a center for comparative studies in the future.

## Conclusion

This report describes the implementation of a new robotic program where the primary measure of success is outcomes. We show that monitoring cholecystectomy, inguinal or ventral hernia data can confirm the quality of the program before expansion and moving forward to more complex procedures. Our methodology is very simple, as we used the standard measures of a surgical practice, which included patient reported outcomes. The model we created should be used for future programs, especially with the new robotic platforms coming to the market.

## Supplementary Information

Below is the link to the electronic supplementary material.

Supplementary file1 (DOCX 102 kb)

## References

[1] Sheetz KH, Claflin J, Dimick JB (2020). Trends in the adoption of robotic surgery for common surgical procedures. JAMA Netw Open.

[2] Crist DW, Gadacz TR (1993). Complications of laparoscopic surgery. Surg Clin N Am.

[3] Palmer KJ, Lowe GJ, Coughlin GD (2008). Launching a successful robotic surgery program. J Endourol.

[4] Zender J, Thell C (2010). Developing a Successful Robotic Surgery Program in a Rural Hospital. AORN J.

[5] Patel VR (2006). Essential elements to the establishment and design of a successful robotic surgery programme. Int J.

[6] Luthringer T, Aleksic I, Caire A, Albala DM (2012). Developing a successful robotics program. Curr Opin Urol.

[7] Dindo D, Demartines N, Clavien PA (2004). Classification of surgical complications: a new proposal with evaluation in a cohort of 6336 patients and results of a survey. Ann Surg.

[8] McCaffery M, Beebe A (1989) Pain: Clinical Manual for Nursing Practice

[9] Quinn J, v., Drzewiecki AE, Stiell IG, Elmslie TJ, (1995). Appearance scales to measure cosmetic outcomes of healed lacerations. Am J Emerg Med.

[10] Breitenstein S, Nocito A, Puhan M (2008). Robotic-assisted versus laparoscopic cholecystectomy: outcome and cost analyses of a case-matched control study. Ann Surg.

[11] Kakiashvili E, Bez M, Abu Shakra I (2021). Robotic inguinal hernia repair: Is it a new era in the management of inguinal hernia?. Asian J Surg.

[12] Gonzalez A, Escobar E, Romero R (2017). Robotic-assisted ventral hernia repair: a multicenter evaluation of clinical outcomes. Surg Endosc.

[13] Balachandran B, Hufford TA, Mustafa T (2017). A comparative study of outcomes between single-site robotic and multi-port laparoscopic cholecystectomy: an experience from a tertiary care center. World J Surg.

[14] Kudsi OY, Castellanos A, Kaza S (2017). Cosmesis, patient satisfaction, and quality of life after da Vinci Single-Site cholecystectomy and multiport laparoscopic cholecystectomy: short-term results from a prospective, multicenter, randomized, controlled trial. Surg Endosc.

[15] Lee SR, Kim HO, Shin JH (2019). Clinical outcomes of single-incision robotic cholecystectomy versus conventional 3-port laparoscopic cholecystectomy. Can J Surg.

[16] Su WL, Huang JW, Wang SN, Lee KT (2017). Comparison study of clinical outcomes between single-site robotic cholecystectomy and single incision laparoscopic cholecystectomy. Asian J Surg.

[17] Pietrabissa A, Pugliese L, Vinci A (2016). Short-term outcomes of single-site robotic cholecystectomy versus four-port laparoscopic cholecystectomy: a prospective, randomized, double-blind trial. Surg Endosc.

[18] Balaphas A, Buchs NC, Naiken SP (2017). Incisional hernia after robotic single-site cholecystectomy: a pilot study. Hernia.

[19] Abdelmoaty WF, Dunst CM, Neighorn C (2019). Robotic-assisted versus laparoscopic unilateral inguinal hernia repair: a comprehensive cost analysis. Surg Endosc.

[20] Prabhu AS, Carbonell A, Hope W (2020). Robotic inguinal vs transabdominal laparoscopic inguinal hernia repair the RIVAL randomized clinical trial. JAMA Surg.

[21] Waite KE, Herman MA, Doyle PJ (2016). Comparison of robotic versus laparoscopic transabdominal preperitoneal (TAPP) inguinal hernia repair. J Robot Surg.

[22] Olavarria OA, Bernardi K, Shah SK (2020). Robotic versus laparoscopic ventral hernia repair: multicenter, blinded randomized controlled trial. The BMJ.

[23] Misiakos EP, Patapis P, Zavras N (2015). Current trends in laparoscopic ventral hernia repair. J Soc Laparoendosc Surg.

[24] Lindström P, Rietz G, Everhov ÅH, Sandblom G (2021). Postoperative pain after robot-assisted laparoscopic ventral hernia repair. Front Surg.

