# Supplementary material for: Implementation of robotic surgery in Dubai: a focus on outcomes
Source: J Robot Surg. 2022 Apr 19;17(1):169–76. doi: 10.1007/s11701-022-01407-8 (PMC9939485; doi:10.1007/s11701-022-01407-8)
Supplement: Supplementary file 1 — Supplementary file1 (DOCX 102 kb) [file 11701_2022_1407_MOESM1_ESM.docx]

Supplemental Appendix:

Special Privileges in Robotic Surgery (DaVinci Surgical Platform) require:

i. Core Privileges in the surgeon’s specialty.

ii. Possession of endoscopic privileges for the procedure(s) to be performed robotically.

iii. Completion of online module and receipt of certificate for the appropriate DaVinci surgical system.

iv. Completion of simulation training on the Mimic “backpack” simulator.

1. Completion, with a score of 80 or higher, of 12 mandatory simulation Mimic modules

v. Attendance at hands on training practicum (8 hrs minimum, with 3 hrs personal time on the system) in use of DaVinci platform or submission of a residency equivalency letter, along with a case log documenting participation in at least 10 cases as bedside assistant and 20 cases as primary console surgeon (recent residency/fellowship graduates only).

vi. Observation of at least 2 clinical cases, utilizing the DaVinci platform, in the surgeon’s specialty.

vii. 3 proctored patient uses, in surgeon’s specialty, of DaVinci platform.

viii. Proctor evaluation on robotic competencies using established criteria. If surgeon not deemed fully competent, proctoring will continue until competence satisfactorily demonstrated in 3 consecutive cases. FPPE forms and Robotic Surgery Proctor Evaluation Forms will be completed for 3 proctored cases.

ix. Upon satisfactory completion of proctoring period (as defined in e-viii), the Department Chairman may grant the surgeon Special Privileges in Robotic Surgery.

x. Department Chairman may wave requirements e-iv through 2-ix for surgeons who, based on documented recent DaVinci platform experience and competence at another hospital, would qualify to be credentialed as a Robotics Proctor at NYPBMH. FPPE and Robotic Surgery Proctor Evaluation forms will be completed for the surgeon’s first 3 robotic cases.

xi. An average of at least 24 robotic cases should be performed during each 2 year reappointment cycle to maintain unrestricted Special Privileges in Robotic Surgery. If volume criteria are not met the surgeon has to complete simulation training, attend a hands-on practicum, and case observation and proctoring as detailed in eiii-ix.

Other robotic surgery considerations:

1. Credentialing as Robotics Proctor:

a. Fulfill criteria e-i and e-ii above.

b. Have attended hands on training practicum (8 hrs minimum); OR be a certified proctor from Intuitive Surgical; OR serve as in-house DaVinci platform proctor at another hospital.

c. Have performed 30 un-proctored cases on DaVinci platform.

d. Must perform at least 25 robotic cases per year.

2. Credentialing as Robotics Surgical Assistant (attending surgeons):

a. Fulfill criterion e-i above.

b. Have attended a hands-on training practicum (4 hrs minimum).

c. Surgical assisting role only applies to bedside assistant function; does not allow assistant to operate surgical console.

| Supplemental Table: Complications |  |  |  |  |  |  |  |  |  |
| --- | --- | --- | --- | --- | --- | --- | --- | --- | --- |
|  |  | Number | Complications according to the Clavien-Dindo degree of severity | | | | | Overall complications | |
|  |  |  | I | II A | II B | III | IV |  |  |
|  |  |  |  |  |  |  |  |  |  |
|  |  |  |  |  |  |  |  |  |  |
| Laparoscopic |  | 33 | 2 | 0 | 0 | 0 | 0 |  | 2 |
| Robotic |  | 30 | 0 | 0 | 0 | 0 | 0 |  | 0 |
| Single Site |  | 40 | 4 | 0 | 0 | 0 | 0 |  | 4 |
|  |  |  |  |  |  |  |  |  |  |
|  |  |  |  |  |  |  |  |  |  |
|  |  |  |  |  |  |  |  |  |  |
| Laparoscopic |  | 78 | 0 | 0 | 0 | 0 | 0 |  | 0 |
| Robotic |  | 68 | 0 | 0 | 0 | 0 | 0 |  | 0 |
|  |  |  |  |  |  |  |  |  |  |
|  |  |  |  |  |  |  |  |  |  |
|  |  |  |  |  |  |  |  |  |  |
| Laparoscopic |  | 17 | 0 | 0 | 0 | 0 | 0 |  | 0 |
| Robotic |  | 19 | 0 | 0 | 0 | 0 | 0 |  | 0 |
| Open |  | 19 | 0 | 0 | 0 | 0 | 0 |  | 0 |
